# The negative effect of wood ant presence on tick abundance

**DOI:** 10.1186/s13071-018-2712-0

**Published:** 2018-03-15

**Authors:** Silvia Zingg, Patrick Dolle, Maarten Jeroen Voordouw, Maren Kern

**Affiliations:** 1 School of Agricultural, Forest and Food Sciences, Bern University of Applied Sciences, 3052 Zollikofen, Switzerland; 2 Institute of Biology, University of Neuchâtel, 2000 Neuchâtel, Switzerland

**Keywords:** Ants, Biological pest control, Ecosystem services, *Formica polyctena*, Ixodidae, Ticks

## Abstract

**Background:**

Ticks and tick-borne pathogens are a global problem for the health of humans and their livestock. Wood ants are important ecosystem engineers in forests worldwide. Although both taxa are well studied, little is known about their interactions under natural conditions. The purpose of the present field study was to test whether European red wood ants (*Formica polyctena*) influence the abundance of *Ixodes* tick populations in temperate forests.

**Methods:**

Data collection took place in 130 sampling plots located at 26 ant nest sites paired with 26 control sites in northwestern Switzerland. At each sampling plot, tick abundance, ant abundance, ant nest volume and habitat variables (describing litter, vegetation and microclimate) were measured. We used linear mixed-effect models to analyze the abundance of questing ticks as a function of ant abundance and habitat variables.

**Results:**

Ant nest volume, rather than the presence of ants, had a significant negative effect on tick abundance. The number of ticks decreased from 11.2 to 3.5 per 100 m^2^ if the volume of the adjacent ant nest increased from 0.1 m^3^ to 0.5 m^3^. Additionally, high vegetation cover and litter depth had negative and positive relationships with tick abundance, respectively.

**Conclusions:**

We showed that the number of questing ticks was negatively correlated with the size of red wood ant nests. Further studies are needed to identify the mechanisms that drive the relationship. Possible mechanisms include the repellent effect of ant formic acid, and the predatory behavior of wood ants. The present field study suggests that red wood ants provide a new ecosystem service by reducing the local abundance of *Ixodes* ticks.

**Electronic supplementary material:**

The online version of this article (10.1186/s13071-018-2712-0) contains supplementary material, which is available to authorized users.

## Background

Ticks are blood-feeding arthropods that vector many different pathogens to a wide range of vertebrate hosts. They are among the most important ectoparasites in veterinary medicine [1] and tick-borne diseases (TBDs) have become prominent public health issues. TBDs such as Lyme borreliosis (LB) or tick-borne encephalitis (TBE) have increased in the last decade [2]. In Western Europe, the hard tick *Ixodes ricinus* is the most important vector of LB and TBE. *I. ricinus* is a three-host tick: the larvae and nymphs feed on a wide variety of vertebrate hosts, such as birds and small and large mammals, whereas the adult ticks exclusively parasitize large mammals such as deer [3, 4]. The abundance of ticks, particularly infected nymphs, is key in determining tick-borne disease risk [2]. Climatic factors (e.g. temperature and humidity) and habitat characteristics, such as vegetation structure or host presence can influence the abundance of ticks [5, 6]. While concerns about the application of chemical acaricides are growing, biological control is becoming an increasingly attractive approach for tick management [7]. Different biocontrol agents such as pathogens (bacteria, fungi), parasitoids (nematodes), or predators can have direct negative effects on tick abundance [8–11]. In addition, predators or competitors of important tick hosts such as rodents may indirectly reduce tick numbers by reducing the host density or activity [12, 13].

Red wood ants (the *Formica rufa* group) are mound building, social insects, that are ubiquitous in European forest ecosystems. The nests are large, conspicuous, dome-shaped mounds of grass, twigs, or conifer needles, and wood ant colonies can include several spatially separated but socially connected nests [14]. Red wood ants can reach high densities and their impact extends over several trophic levels and ecosystem processes. Their ecological roles include: altering the soil composition and nutrient flow via mound building, dispersing seeds, engaging in mutualistic relationships with aphids, preying on invertebrates, and competing with other predators including insectivorous birds and other ant species. Red wood ants are therefore recognized as keystone species, ecosystem engineers, and biocontrol agents against forest pests [15–19]. Most wood ant species are considered ‘near threatened’ by the IUCN [20]. Although a lot is known about the ecological importance of red wood ants, studies describing the relationship between red wood ants and ticks are rare [21]. Ants have direct effects on tick abundance via predation and the repellent effect of formic acid, and they have indirect effects on tick abundance by influencing host availability [12, 22, 23]. In most cases, however, the role of ants in reducing tick populations is not clear; field studies are needed because results from laboratory studies are hard to extrapolate to natural conditions [22].

In this study, we examined the effect of the presence of the European red wood ant *Formica polyctena* on the abundance of ticks from the genus *Ixodes* in northern Switzerland. We tested two hypotheses: (i) the abundance of ticks is lower in the presence of an *F. polyctena* nest and (ii) the abundance of ticks is lower when the abundance of *F. polyctena* is high. In addition, a small tick exposure experiment was conducted to observe the interactions between *F. polyctena* and different tick life stages.

## Methods

### Study site

The study was conducted in the northwestern part of Switzerland in the cantons Basel-Land and Solothurn. The region is part of the Jura Mountains at an altitude of 389 to 989 m above sea level. Settlements, forests and agricultural areas are the dominant land cover types in this densely populated region. Forests are typically mixed stands of beech (*Fagus sylvatica*), Norway spruce (*Picea abies*) and silver fir (*Abies alba*) and contain both even-aged and uneven-aged planter stands.

### Experimental design

**Fig. 1:**
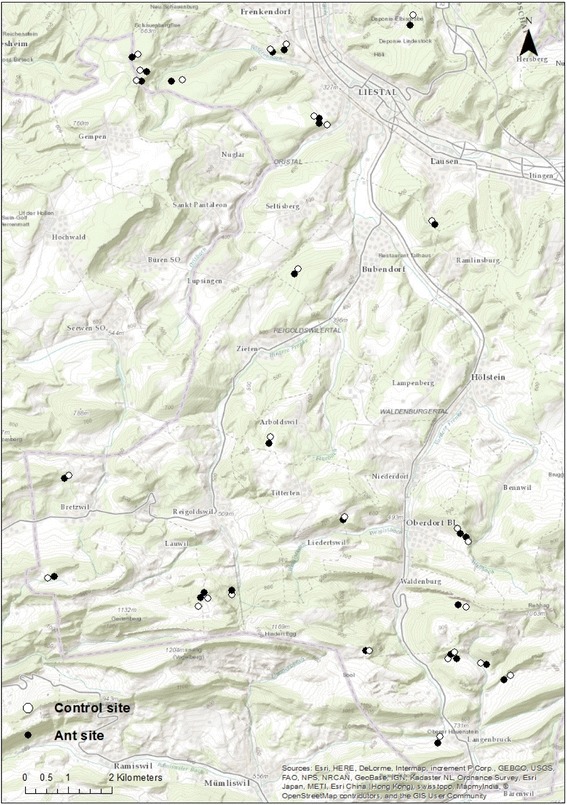
Study region. Map showing the study area in the Jura Mountains in northwestern Switzerland. Ant nest sites of *Formica polyctena* (black) and control sites (white) were always paired and spaced 100–300 m apart. Sources: Esri, HERE, DeLorme, Intermap, increment P Corp., GEBCO, USGS, FAO, NPS, NRCAN, GeoBase, IGN, Kadaster NL, Ordnance Survey, Esri Japan, METI, Esri China (Hong Kong), swisstopo, MapmyIndia, © OpenStreetMap contributors, and the GIS User Community

All wood ant nests in the study region had been systematically mapped and determined to species level by a wood ant project in 2015/2016 [24]. In this project, a total of 1095 ant nests were found for a number of different ant species. We decided to study *F. polyctena* for the following reasons: (i) this ant species is common; (ii) its habitat overlaps with *I. ricinus*; and (iii) a previous study suggested that *F. polyctena* had negative effects on tick burdens on vertebrate hosts (hares) [25]. For our study, we randomly selected 26 *F. polyctena* nest sites. Each nest site was paired with a control site where *F. polyctena* was absent (Fig. 1). The control sites were randomly generated using ArcGIS 10.2.2 in a spatial buffer of 100–200 m around the nest sites. We checked the control sites in the field for the presence of *F. polyctena* and changed the position of the control site if ants were detected. All control sites met the following four criteria: (i) distance of 100–300 m to nest site; (ii) absence of *F. polyctena*; (iii) forest type similar to the nest site; and (iv) distance to forest edge similar to the nest site. To check the last criterion, the mean minimal distance from each site to the forest edges or roads was computed using ArcGIS 10.2.2. There was no significant difference in the distance to forest edges or roads between the ant sites and the control sites (t-test: *P* = 0.56).

### Data collection

**Fig. 2:**
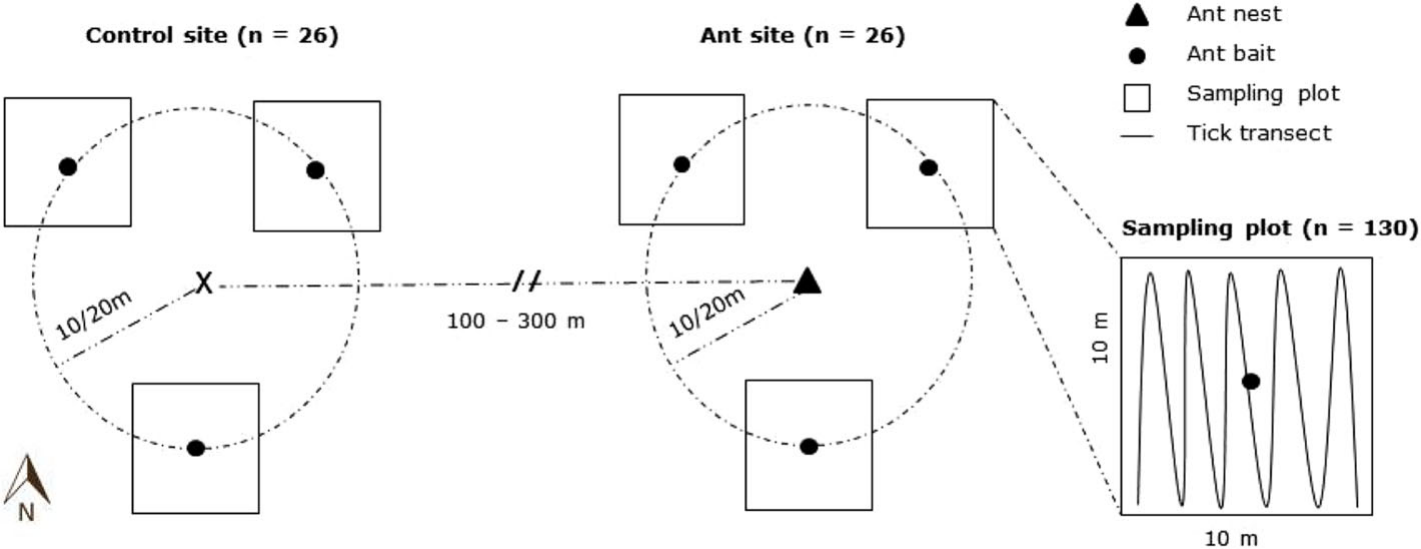
Sampling design. Twenty six ant nest sites of *Formica polyctena* and 26 paired control sites were selected. The center of the ant site corresponded to the ant nest whereas the center of the control site was randomly generated. Data collection took place in the sampling plots (*n* = 130), which were placed at a distance of 10 m or 20 m from the center of the site. Collection of questing *Ixodes* ticks took place along a transect line in all sampling plots. Ant baits were used to determine the abundance of *Formica polyctena* ants

Data collection at each ant site and control site took place in three sampling plots of 10 × 10 m. From the center of each site, sampling plots were oriented in three compass directions: 60°, 180°, and 300° (Fig. 2). This orientation ensured that at least two of the three sampling plots were located in the forest, because ant nests were often found at southern forest edges. Sampling plots that fell outside the forest, in grasslands or other land cover types, were not considered. Wood ants mainly forage within a radius of 50 m around their nests [26]. The sampling plots were therefore placed at distances of 10 m or 20 m from the center of the site. We collected data in 130 sampling plots at 26 ant sites (*n* = 65 sampling plots) and 26 control sites (*n* = 65 sampling plots). Of the 130 sampling plots, 68 and 62 were placed at a distance of 10 m and 20 m from the center of the site, respectively. The sampling sequence for the sites was randomized. Two to six sites (depending on the weather conditions and the distances between the sites) were sampled per day, randomly alternating between the ant sites and the control sites. Sampling took place between May and July 2016, when tick density is supposed to be high [27, 28] and when the weather was suitable: moderate temperature (mean temperature of 20 °C) and no rain [6]. We dragged a 1 m^2^ white linen sheet attached to a wooden pole over the soil and the vegetation to collect questing ticks [29]. A predefined transect (length = 100 m) was walked and the sheet was checked for nymphs and adult ticks every 10 m. Larvae were not included because they are highly clustered in the places where adult females lay their clutch of eggs [30]. The linen sheet was changed after each site, to avoid accumulating tick-repellent substances such as formic acid produced by the ants. All ticks were collected and stored. At the Institute of Parasitology at the University of Zürich, one tick per sampling plot was randomly selected for identification. Selected ticks were identified as *Ixodes ricinus* either morphologically (*n* = 6 adults) [31], or genetically (*n* = 6 nymphs). Nymphs were genetically identified by PCR/sequencing as described in Karger et al. [32]. All other nymphs were identified to genus level (not to species level) as *Ixodes* ticks (*n* = 109 nymphs). We assume that most of the collected ticks were *Ixodes ricinus*, as it is the most abundant *Ixodes* species in Switzerland [33].

Ant abundance was estimated by using a bait consisting of one tablespoon of tuna on a circular sheet of paper (Ø = 14 cm). The number of ants attracted to the bait can be used as a proxy for the ant population density in the surrounding area [34]. Tuna baits were placed in the center of every sampling plot for ~ 20 min and all ants found on the paper were counted. The height and the diameter of each ant mound were measured to calculate the ant nest volume, which is an indirect measure of the ant population size [35, 36]. The following habitat variables were measured within the sampling plots and were included in the analysis: litter depth (cm), litter composition (% of needles), moss cover (%), low vegetation (< 0.4 m) cover (%), high vegetation (> 0.4 m) cover (%), canopy cover (%), and the saturation deficit (SD; mmHg). To calculate the SD, we measured air temperature and relative humidity with a thermo-hygrometer (Hygropalm HP21, Rotronic, Bassersdorf, Switzerland) at 0 m, 0.5 m and 1 m above the ground at two opposing edges in each sampling plot [37]. Mean temperature and mean humidity (per sampling plot) were subsequently used to calculate the SD, which is known to influence tick questing activity [5, 38]. An overview on all variables can found in Table 1. The complete data set is available in Additional file 1: Table S1 and S2.

**Table 1:** Summary statistics are shown for the tick, ant, habitat and climate variables included in the statistical analysis

Parameter	Mean ± SE	Range
Tick nymphs (*n*)	15.7 ± 1.3	0–69
Tick adults (*n*)	0.2 ± 0.1	0–4
Ant nest presence (0/1)		
Ant nest volume (m^3^)	0.18 ± 0.02	0.002–0.8
Ant abundance (*n*)	11.4 ± 2.0	0–120
Litter depth (cm)	3.4 ± 0.2	0.1–12.5
Litter composition (% needles)	24.8 ± 2.2	0–100
Moss cover (%)	7.4 ± 0.8	0–60
Low vegetation cover (%)	29.3 ± 2.1	0–90
High vegetation cover (%)	38.2 ± 2.3	0–100
Canopy cover (%)	70.3 ± 1.8	0–95
Temperature^a^ (°C)	20.3 ± 0.3	13–30
Humidity^a^ (%)	72.1 ± 0.8	42–95

^a^Temperature and humidity were used to calculate the saturation deficit

*Abbreviation*: *SE* standard error

### Tick exposure experiment

To test the hypothesis that predation may be responsible for the negative effect of wood ants on tick abundance, we conducted a small on-site field experiment. We repeatedly exposed *Ixodes* ticks to *Formica polyctena* ants (5 trials with nymphs and 5 trials with adults) and recorded the behavior of the ants. The ticks were either fed (i.e. engorged with blood; 3 adults), or unfed (2 adults and 5 nymphs). The engorged ticks were collected from farm animals (horses and dogs) in the study region, and the unfed ticks were collected using the dragging method in the forest. For the experiment, the ticks were placed on a white paper sheet for 10 min. The paper sheets were placed on the ground at locations of high ant density (ant streets) within a radius of 5–10 m to the ant nest. We observed the interactions between the wood ants and the ticks (e.g. scanning, grabbing or aggression). The results of this small experiment were included in the discussion but were not analyzed statistically due to the small sample size (*n* = 10 observations).

### Statistical analysis

The aim of the statistical analysis was to describe the relationship between the abundance of ticks and the abundance of wood ants, while taking habitat variables into account. The response variable was the total number of ticks (adults and nymphs). The number of ticks was square root-transformed to improve the model fit and the normality of the residuals. Ant and habitat variables were used as explanatory variables and their correlations were assessed using Pearson’s correlation coefficient. Habitat and ant variables were not correlated (Pearson’s correlation coefficient <  0.50). Linear mixed-effects models were used to analyze the relationship between ticks, ants and habitat variables (R package *lme4*). Although the sampling plots were located at two different distances (10 m and 20 m to the center), distance had no significant effect on the number of ticks and was therefore removed from the full model. The following full model was fitted:

$$ \mathrm{lmer}\left(\mathrm{sqrt}\ \left(\mathrm{Number}\ \mathrm{of}\ \mathrm{ticks}\right)\sim \mathrm{Nest}\ \mathrm{presence}+\mathrm{Nest}\ \mathrm{volume}+\mathrm{Ant}\ \mathrm{abundance}+\mathrm{LD}+\mathrm{LC}+\mathrm{MC}+\mathrm{LV}+\mathrm{HV}+\mathrm{CC}+\mathrm{SD}+\left(1|\mathrm{Pair}/\mathrm{Site}\right)\right) $$

where LD is litter depth, LC is litter composition, MC is moss cover, LV is low vegetation cover, HV is high vegetation cover, CC is canopy cover and SD is saturation deficit.

In the full model, the 130 sampling plots were the sampling units and the random effects structure included the 52 sites (“Site”) nested within the 26 site pairs (“Pair”). Three different ant variables were used as explanatory variables: (i) ant nest presence; (ii) ant nest volume; and (iii) ant abundance. Ant nest presence was defined as 1 and 0 for sampling plots at ant sites and control sites, respectively. We calculated the volume of each ant nest by applying the formula for a cone to the height and diameter of each nest [39]. Sampling plots at control sites were assigned an ant nest volume of zero. Ant abundance was the number of ants counted in the sampling plots using bait. For the subset of the 65 sampling plots near ant nests, the ant nest volume and number of ants per sampling plot were correlated (Pearson’s correlation coefficient = 0.59, *df* = 63, *P* <  0.001). After fitting the full model, we used the dredge function (R Package *MuMIn*) to fit all possible candidate models. We used an information-theoretic model selection procedure to evaluate the models [40]. Models were ranked according to their corrected Akaike information criterion (AICc), and the best models have the lowest AICc scores. The difference in AICc between the best model and a model of interest (∆ AICc) is used to calculate the weight or support for each model. These model weights are used to calculate the model-averaged parameter estimates for a set of models. In our case, the model-averaged parameter estimates were calculated for the subset of candidate models with ∆ AICc < 4 (R Package *MuMIn*). The sum of the model weights (∑w_i_) was calculated for each parameter. Parameters with ∑w_i_ > 0.5 and significant *P*-values were defined as good predictors for tick abundance [40]. All statistical analyses were conducted in R version 3.2.5 [41].

## Results

We collected a total of 2062 ticks at our sites: 28 adult ticks and 2034 nymphs. Of the 28 adult ticks, 7 and 21 were collected at ant nest sites and control sites, respectively. Of the 2034 nymphs, 926 and 1108 were found at ant nest sites and control sites, respectively. Ticks were found in 121 of the 130 sampling plots and were identified (one tick per sampling plot) as *Ixodes* sp. (see Data collection). The mean ± SD tick abundance was 16 ± 16 per sampling plot, with a maximum count of 70 ticks. On average, 14 ± 16 ticks were found at ant nest sites and 17 ± 15 at control sites. At the 26 ant nest sites, the mean number of ticks was 13 ± 15 for sampling plots located at 10 m and 15 ± 17 for sampling plots located at 20 m from the ant nest (see Additional file 2: Figure S1).

Baiting confirmed ant activity in 59 of the 65 sampling plots at ant nest sites and in 4 of the 65 sampling plots at control sites. We counted a total of 1472 *F. polyctena* individuals: 1458 were found in the ant nest sites and 14 were found in the control sites. At the ant nest sites, the mean ± SD abundance of *F. polyctena* was 23 ± 28 ants per sampling plot, with a maximum count of 120 ants. The mean ant nest volume was 0.18 ± 0.18 m^3^. Other ant species were rarely observed in the sampling plots at the *F. polyctena* nest sites (*n* = 4 sampling plots) and the control sites (*n* = 9 sampling plots) and included: *Formica fusca* (*n* = 2 sampling plots), *Camponotus ligniperda* (*n* = 4 sampling plots), *Myrmica* sp. (*n* = 6 sampling plots), and *Lasius* sp. (*n* = 3 sampling plots).

### The influence of ants on tick abundance

**Fig. 3:**
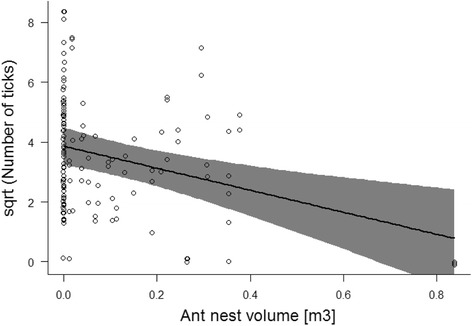
Model-averaged predictions of tick abundance. The significant negative relationship between the number of *Ixodes* ticks and the ant nest volume. Shown are model-averaged predictions from the linear mixed models with 95% Bayesian credible intervals

The best model for explaining tick abundance contained the variables: litter depth, high vegetation cover and ant nest volume (Table 2). Model averaging revealed that ant nest volume and high vegetation cover had significant negative relationships with tick abundance, whereas litter depth had a significant positive relationship with tick abundance (Table 3). If the volume of an ant nest increased from 0.1 m^3^ to 0.5 m^3^, the tick abundance decreased from 11.2 to 3.5 ticks per sampling plot. Overall, ant nest volume was the best ant-related predictor for tick abundance. The negative relationship was influenced by one particularly large ant nest with a volume of 0.8 m^3^ (see Fig. 3). When this nest was excluded from the dataset, the negative effect of ant nest volume on tick abundance decreased but ant nest volume still had a high importance (see Additional file 2: Table S3). When presence/absence of the ant nest was included as the only ant-related predictor (i.e. the volume of the ant nest and ant abundance were removed from the model), the negative effect of ants on tick abundance was less pronounced. Nevertheless, ant nest presence was still in the best model and of importance (see Additional file 2: Table S4). Finally, we calculated the variance components for the following three random factors in our sampling design: pairs (*n* = 26 pairs), site (*n* = 52 sites), and sampling plots (*n* = 130 sampling plots). The variance component was highest for pairs (45.8%), lowest for site (17.3%), and intermediate for sampling plot (36.9%). This result indicates that most of the variation in tick abundance occurred between the 26 pairs, due to environmental differences and temporal differences in when these pairs were sampled. The result also indicates that our approach of pairing ant nest sites with control sites was effective in reducing the variance in tick abundance (i.e. the level site had the lowest variance in tick abundance).

**Table 2:** Model selection table. Model selection table that includes the six best models is shown. Models with ∆AICc > 4 are not shown. The response variable was the total number of *Ixodes* ticks in a sampling plot. The best models included two ant-related explanatory variables, ant nest volume and ant nest presence, and two environmental variables, high vegetation cover and litter depth

No.	Intercept	High veg. Cover	Litter depth	Ant nest presence	Ant nest volume	*df*	logLik	∆AICc	Akaike weight
1	3.83	-0.02	0.19		-3.64	7	-235.4	0.0	0.38
2	3.20		0.19		-3.59	6	-237.1	1.2	0.21
3	4.49	-0.02			-3.72	6	-237.6	2.3	0.12
4	3.79	-0.02	0.19	+	-3.92	8	-235.4	2.3	0.12
5	3.87				-3.67	5	-239.0	2.8	0.09
6	3.15		0.19	+	-4.01	7	-237.1	3.4	0.07

**Table 3:** Model-averaged parameter estimates. Model-averaged parameter estimates calculated over the model set in Table 2. The response variable was the total number of *Ixodes* ticks in a sampling plot. The explanatory variables included: ant nest volume, ant nest presence, high vegetation cover, and litter depth. The sum of model weights (∑w_i_) indicates the relative importance of each parameter

	Estimate	Std. Error	*Z*-value	*P*-value	∑wi
(Intercept)	3.72	0.55	6.70	< 0.001	1.00
Ant nest volume	-3.70	1.11	3.30	< 0.001	0.99
Litter depth	0.19	0.07	2.83	< 0.001	0.73
High vegetation cover	-0.02	0.00	3.61	< 0.001	0.62
Ant nest presence	0.14	0.37	0.37	0.71	0.25

### Tick exposure experiment

In the tick exposure experiment, no interactions were observed between *F. polyctena* ants and nymphal ticks. The ants did not interact with or attack the nymphs, but just walked over them (*n* = 5 trials). However, we did observe interactions between ants and adult ticks. Ants always attacked adult ticks or examined them using their antennae and mandibles (*n* = 5 trials), regardless of the tick engorgement status. In addition, the ants removed the engorged adult ticks (*n* = 3 trials).

## Discussion

Our study is the first field study to investigate the relationship between the abundance of red wood ants and the abundance of questing *Ixodes* ticks. We confirmed our hypothesis that the abundance of questing *Ixodes* ticks is lower in the presence of *F. polyctena* nests. We tested the effect of three ant-related variables on tick abundance and found that ant nest volume was the most important. The number of questing *Ixodes* ticks (mainly nymphs) decreased significantly as the volume of the ant nest increased. A reduction in the density of questing nymphs can reduce the risk of tick-borne diseases, as nymphs have the highest impact on disease transmission to humans [11, 42].

Nest mound volume was a more important predictor of the negative impact of wood ants on the abundance of *Ixodes* ticks than our estimates of ant abundance, which were based on a time-limited baiting method. Nest mound volume is a proxy for ant population size and ant foraging activity [36, 43], but is also influenced by environmental factors such as soil composition and temperature [44]. In polydomous species such as *F. polyctena*, the colony structure may influence the effect on ticks and future studies should investigate the spatial component of the negative effect of wood ants on ticks.

We emphasize that our correlational study does not allow us to determine the mechanism underlying the observed negative relationship between wood ants and ticks. Possible explanations include predation, as more than 30 ant species, including *F. polyctena*, are known to occasionally prey on ticks [22, 25]. Our small exposure experiment confirmed that red wood ants potentially prey on ticks. The developmental stage influenced the interaction; *Formica polyctena* ants did not interact with nymphal ticks, but carried off engorged adult ticks, which are probably an easier and more attractive prey than unfed ticks [22]. However, scientific evidence is rare and the predatory behavior of wood ants on ticks is debated [21]. The only field studies that have shown that ants are efficient predators of ticks (e.g. [45, 46]) and effectively reduce tick abundance were conducted with the red fire ant *Solenopsis invicta* [12]. In general, wood ants are generalist predators and their feeding on ticks seems to be sporadic [7]. The suitability of such generalist predators to control tick populations has been questioned [7, 47]. Nevertheless, it is known that wood ants can influence the populations of predatory arthropods (e.g. [48, 49]) and that ant chemical cues play an important role [50]. Several studies have demonstrated that ant formic acid repels ticks [22, 23, 25]. Ant formic acid is a volatile organic acid that is produced by ants for defense or trail marking. It acts as chemical weapon and general alarm signal and hundreds of ants can simultaneously release it [51]. Thus, the surrounding of ant nests is covered with ant formic acid, which in turn, may negatively affect tick abundance.

In addition to the direct effects of wood ants on ticks due to predation and/or formic acid, it is also possible that wood ants influence the presence of tick hosts (e.g. small mammals). The abundance and activity of vertebrate hosts are important predictors for tick abundance and tick-borne disease risk [52, 53]. It has been shown that predators such as foxes can influence the behavior of tick hosts such as wood mice and have cascading effects on tick abundance and tick-borne disease risk [13]. Some ant species, such as the red fire ant (*Solenopsis invicta*), actually prey on small mammals, which appears to have negative cascading effects on small mammal density and tick density [12]. Although red wood ants do not prey on small mammals, the latter may still reduce their activity [54] in proximity to ant nest mounds.

The abundance of ticks is also influenced by other biotic and abiotic habitat variables (e.g. vegetation, temperature, or humidity) [5, 6]. In our study, litter depth and high vegetation cover were correlated with the number of ticks. In general *Ixodes* ticks are present in areas with a good cover of vegetation and litter, because they are sensitive to desiccation and require a high relative humidity [4, 5]. Ticks hide in the litter to avoid dehydration when temperatures are high [5, 27], which explains the positive effect of litter depth on tick abundance. Vegetation cover is an important factor, as it influences the microclimate, and allows questing ticks to wait for passing hosts. There are two different explanations for the slight negative effect of high vegetation cover on tick abundance in our study. One explanation is that ticks avoided plots with high vegetation cover, which seems counterintuitive as in general *I. ricinus* favors forested habitats with a dense herb and shrub layer (e.g. [4]). Another explanation is, that the efficacy of tick sampling was reduced in plots with high vegetation cover, as shown in several studies [55, 56]. Finally we cannot say if tick abundance or detectability was decreased by the high vegetation cover in some sampling plots [57].

## Conclusions

We showed that the presence of red wood ants was negatively associated with the number of questing *Ixodes* ticks. Ant nest volume was the most important ant-related variable and had a strong negative effect on tick abundance. The mechanisms that drive the negative relationship between wood ants and ticks remain unknown. Possible mechanisms include the repellent effect of ant formic acid, and the predatory behavior of the wood ants. Wood ants are known to influence the forest ecosystem and to provide important ecosystem services. Conservation and promotion of wood ants can therefore sustain these functions and may have negative effects on tick abundance. As tick-borne diseases are a prominent public health issue, future studies should explore the role of wood ants in controlling tick abundance.

## Additional files

Additional file 1: **Table S1.** Description of all variables used in the statistical analysis. **Table S2.** Raw data. (XLSX 38.7 kb)

Additional file 2: **Figure S1.** Number of ticks with distance to the center. The number of *Ixodes* ticks per sampling plot is compared between the control sites and the ant nest sites. **Table S3.** Model-averaged parameter estimates of the three most important ecological factors that explain *Ixodes* tick abundance. The data set excluded one ant nest that had a very large volume of 0.8 m^3^. **Table S4.** Model-averaged parameter estimates of the three most important ecological factors that explain *Ixodes* tick abundance. This model selection analysis included only ant nest presence (i.e. the other two ant-related variables were excluded). (DOCX 48.9 kb)
