# Supplementary material for: The negative effect of wood ant presence on tick abundance
Source: Parasit Vectors. 2018 Mar 15;11:164. doi: 10.1186/s13071-018-2712-0 (PMC5852968; doi:10.1186/s13071-018-2712-0)
Supplement: Supplementary file 2 — Figure S1. Number of ticks with distance to the center. The number of Ixodes ticks per sampling plot is compared between the control sites and the ant nest sites. Table S3. Model-averaged parameter estimates of the three most important ecological factors that explain Ixodes tick abundance. The data set excluded one ant nest that had a very large volume of 0.8 m3. Table S4. Model-averaged parameter estimates of the three most important ecological factors that explain Ixodes tick abundance. This model selection analysis included only ant nest presence (i.e. the other two ant-related variables were excluded). (DOCX 48.9 kb) [file 13071_2018_2712_MOESM2_ESM.docx]

## Additional file 2


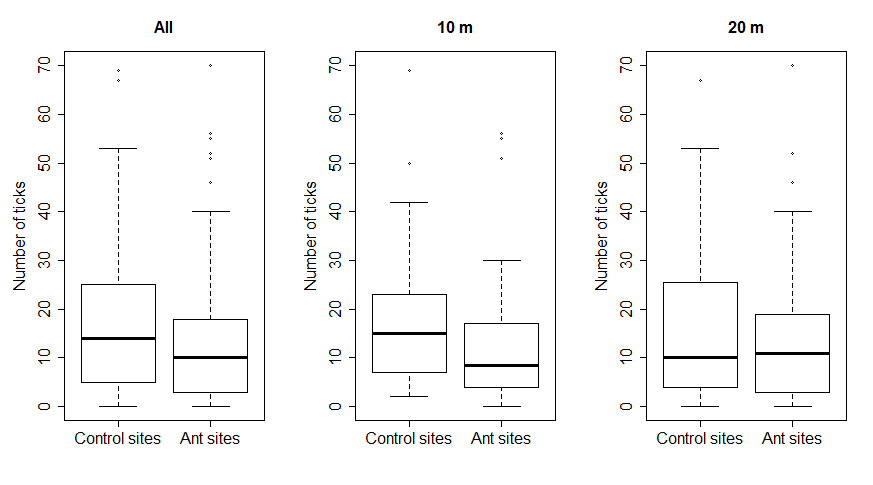


## Figure S1: Number of ticks with distance to the center. The number of *Ixodes* ticks per sampling plot is compared between the control sites and the ant nest sites. The two separate panels refer to whether the sampling plots were placed at distances of 10 m (n = 68 sampling plots) or 20 m (n = 62 sampling plots) from the center of the site. Shown are the medians, quartiles and outliers.

## Analysis without nest volume outlier

To test how one particularly large ant nest, with a volume of 0.8 m^3^, influenced the results, we excluded this nest from the dataset (n = 124 sample plots). In this analysis, ant nest volume was the only ant-related predictor included in the model. The best model for explaining *Ixodes* tick abundance contained the factors litter depth, high vegetation cover, and ant nest volume. Table S1 shows the model-averaged parameter estimates from the conditional average (models with ∆AICc < 4). The sum of model weights (∑w_i_) indicates the relative importance of each parameter.

**Table S3**: Model-averaged parameter estimates of the three most important ecological factors that explain *Ixodes* tick abundance. The data set excluded one ant nest that had a very large volume of 0.8 m^3^. For each parameter, the parameter estimate, its standard error, the z-value, the p-value, and the sum of the model weights (∑wi) are shown.

|  | Estimate | Std. Error | z-value | p-value |  | ∑wi |
| --- | --- | --- | --- | --- | --- | --- |
| (Intercept) | 3.566 | 0.590 | 6.017 | < 0.001 | *** | 1.00 |
| Litter depth | 0.191 | 0.070 | 2.686 | 0.007 | ** | 0.69 |
| High vegetation cover | -0.017 | 0.005 | 3.291 | < 0.001 | *** | 0.38 |
| Ant nest volume | -2.585 | 1.418 | 1.805 | 0.0711 | **.** | 0.94 |

## Analysis of tick abundance using only the presence/absence of nests

To test if ant nest presence per se was associated with *Ixodes* tick abundance, we fitted a model with ant nest presence (absent = 0, present = 1) as the only ant-related predictor (n = 130 sample plots). The best model explaining tick abundance contained the factors litter depth, high vegetation cover, and ant nest presence. Table S2 shows the model-averaged parameter estimates from the conditional average (models with ∆AICc < 4). The sum of model weights (∑w_i_) indicates the relative importance of each parameter.

**Table S4:** Model-averaged parameter estimates of the three most important ecological factors that explain *Ixodes* tick abundance. This model selection analysis included only ant nest presence (i.e. the other two ant-related variables were excluded). For each parameter, the parameter estimate, its standard error, the z-value, the p-value, and the sum of the model weights (∑wi) are shown.

|  | Estimate | Std. Error | z-value | p-value |  | ∑wi |
| --- | --- | --- | --- | --- | --- | --- |
| (Intercept) | 3.691 | 0.598 | 6.150 | < 2e-16 | *** | 1.00 |
| Litter depth | 0.189 | 0.070 | 2.663 | 0.0077 | ** | 0.66 |
| High vegetation cover | -0.017 | 0.005 | 3.618 | 0.0003 | *** | 0.64 |
| Ant nest presence | -0.588 | 0.323 | 1.805 | 0.0711 | **.** | 0.57 |
